# Genome Sequences of Gordonia Phages GrootJr and NovumRegina

**DOI:** 10.1128/mra.00703-22

**Published:** 2022-11-07

**Authors:** Erin L. Doyle, Georgia M. Elliott, Sean J. Hummel, Katelyn A. Jindra, Ashley T. Marsh, Dane M. Bowder

**Affiliations:** a Department of Biology, Doane University, Crete, Nebraska, USA; Loyola University Chicago

## Abstract

Two *Gordonia* bacteriophages, GrootJr and NovumRegina, were discovered, sequenced, and annotated. These phages were isolated from distinct soil and water samples, respectively, on Gordonia terrae 3612. These phages belong to the CR2 subcluster and are similar in genome size and gene content.

## ANNOUNCEMENT

There are more than 14,000 sequenced bacteriophage genomes, with this number rapidly increasing (1). Bacteriophages have the potential to be used as a means of controlling bacterial populations, particularly in medical and bioremediation settings (2, 3). In this study, the genomes of the *Gordonia* phages GrootJr and NovumRegina were sequenced and annotated. These phages were isolated from distinct soil and water samples, respectively, collected at Doane University (Crete, NE) during August 2019. The samples were suspended in peptone-yeast-calcium (PYCa) liquid medium and filtered (0.22-μm pores). Filtrates were inoculated with Gordonia terrae 3612, grown at 30°C for 2 days with agitation, filtered again (0.22 μm), and plated with *G. terrae* in PYCa top agar. Well-isolated plaques were picked and plated with *G. terrae* for three rounds. Transmission electron microscopy (TEM) imaging determined both phages to be siphoviruses. Both were assigned to the lytic CR2 subcluster based on whole-genome comparison (4, 5).

Genomic DNA was extracted from phage lysates using the Wizard DNA cleanup system (Promega). Sequencing libraries were prepared from the phage genomic DNA using a New England Biolabs Ultra II kit with dual-indexed barcoding. The prepared libraries were sequenced on an Illumina MiSeq instrument to obtain single-end, 150-base reads. The coverage and read numbers for each phage are shown in Table 1. The raw reads provided by the sequencer were assembled using Newbler v.2.9 with default settings and in each case, yielded a single phage contig. Consed v.29 was used as described to determine both contigs to be complete and accurate and to possess 3′ 10-base sticky overhangs (6).

**TABLE 1:** Characteristics of phages GrootJr and NovumRegina

Phage name	GC content (%)	Sequence length (bp)	Sequencing coverage (×)	No. of reads	No. of genes	No. of genes with assigned functions	PhagesDB entry^*a*^
GrootJr	65.9	67,176	896	424,189	93^*b*^	36	https://phagesdb.org/phages/GrootJr/
NovumRegina	65.9	67,175	1,014	480,356	92	36	https://phagesdb.org/phages/NovumRegina/

a The PhagesDB page contains TEM and plaque images of the phages.

b The GrootJr GenBank file is missing a gene designated gene 3. Thus, the last gene in the file is numbered as gene 94, but the file contains 93 genes total.

Gene locations were predicted by auto-annotation in DNA Master v.5.23.5 using Glimmer v.3.02b (7) and GeneMark v.3.25 (8). Predicted genes and start sites were verified manually using comparisons to similar phage genes with BLASTp (9), ribosomal binding site (RBS) scores within DNA Master, and Starterator (https://seaphages.org/software). Gene functions were assigned based on comparison to other CR2 and similar phages using Phamerator (10) and PhagesDB (11), along with protein sequence alignments using NCBI BLAST (9) and HHpred (12). Membrane proteins were identified using TMHMM v.2.0 (13) and SOSUI (14, 15). Characteristics of the genome assemblies and annotations are shown in Table 1.

The annotated genomes of GrootJr and NovumRegina share 100.0% nucleotide identity and are 67,176 and 67,175 bases long, with 93 and 92 genes, respectively. The only difference between the genomes is a single-base indel located at position 5329 in GrootJr’s genome. This thymine in the GrootJr genome is not present in NovumRegina’s genome. The deletion accounts for the difference in genome lengths and for the presence of an additional gene, called GrootJr_8, in GrootJr’s genome. This gene, located between base pairs 5182 and 5538, is not called in NovumRegina because the deletion at position 5329 results in the lack of an in-frame stop codon. GrootJr_8 has no assigned function.

### Data availability.

The complete genome sequences for bacteriophages GrootJr and NovumRegina have been deposited at GenBank under accession numbers MT986025 and MT889393, respectively. The raw reads have been deposited in the Sequence Read Archive (SRA) under accession numbers SRX15605399 and SRX15605404 for GrootJr and NovumRegina, respectively.
